# The development of day surgery in China and the effectiveness and reflection of day surgery in ophthalmology-specialized hospitals

**DOI:** 10.1186/s12962-024-00558-9

**Published:** 2024-05-27

**Authors:** Dong Haihan, Zheng Changfei, Lian Hengli, Tang Ning, Zhuo Lezhen, Lin Hui

**Affiliations:** Eye Hospital, Wenzhou Medical University, Wenzhou, 31000 China

**Keywords:** Ambulatory surgery, Medical insurance payment, Medical quality, Health economics

## Abstract

This survey investigates the development of day surgery in China, and analyzes the national policy support, medical service management model, disease types of day surgery, medical insurance payment methods, and the medical service capacity, efficiency, quality and safety, health economics indicators, and patient satisfaction after the implementation of day surgery in a tertiary eye hospital. After more than 20 years of development, China’s day surgery has shown a good development trend. The implementation of day surgery in eye hospitals accounts for more than 70% of elective surgery, and patients, medical institutions, and medical insurance institutions have all achieved good social benefits.

Day surgery refers to a surgical model in which patients complete preoperative examinations before admission, undergo surgery and related treatments on the same day of admission, and are discharged from the hospital [1]. The Chinese Day Surgery Cooperation Alliance (CASA) defines day surgery as the discharge of a patient within 24 h after undergoing a surgical procedure or operation. Day surgery in China has undergone a rapid development stage from clinical independent exploration, spontaneous development by medical institutions, pilot implementation, and government-led development. The results of the seventh national census in 2021 showed that the proportion of people aged 60 and over in China reached 18%, and the trend of population aging has exacerbated the shortage of high-quality medical resources. Vigorously developing day surgery can alleviate the contradiction between supply and demand to a certain extent. Therefore, investigating the development of day surgery in China and a tertiary eye hospital provides a reference for the development of day surgery.

## Survey on the implementation of day surgery nationwide

### Three stages of development of day surgery

China’s day surgery has gone through three stages of development. The first stage was from before 2001 when clinical doctors led the exploration of day surgery. The second stage was from 2001 to 2011 when medical institutions began to develop day surgery. China’s earliest day surgery was first established by the Kwong Wah Hospital in Hong Kong in 2002, and since 2003, day surgery has been gradually carried out in Shanghai, Beijing, Chengdu, Wuhan, and other places [2]. The third stage was from March 2012 to 2014, when the National Health Development Research Center led the establishment of the China Day Surgery Alliance (CASA). The alliance organized and popularized day surgery systematically. The fourth stage was from 2015 to the present, a rapid development stage led by the government. In 2019, the number of national day surgery pilot hospitals reached 129. The National Tertiary Public Hospital Performance Evaluation, which is used as an evaluation and incentive for hospitals to improve the quality and efficiency of medical services, and promote the development of medical and health undertakings, takes the proportion of day surgery to elective surgery as a monitoring indicator. The results of the national examination in 2020 were announced, showing that the number of day surgery patients in tertiary public hospitals increased by 6.44% compared with 2019, and the proportion of day surgeries to elective surgeries was 10.85%, an increase of 1.93% points compared with 2019. There are currently 618 medical institutions carrying out day surgery in China, and nearly 70% of tertiary public hospitals have carried out day surgery [3].

### National policy support for day surgery

After 2015, the country has issued a series of policy supports. In March 2015, the National Health Commission and the State Administration of Traditional Chinese Medicine issued the “Notice on Issuing the Action Plan to Further Improve Medical Services“ [4], requiring hospitals to gradually implement day surgery under the conditions of having minimally invasive surgery and anesthetic support, selecting small and medium elective surgeries that previously required hospitalization, with a single diagnosis, clear clinical pathway, and controllable risks. In February 2017, the “Notice on Issuing the Work Plan for Deepening the Implementation of the Action Plan to Further Improve Medical Services in 2017“ [5] required that under the premise of ensuring medical quality and safety, all the medical institutions should promote the pilot work of day surgery in tertiary hospitals, explore the transformation of some inpatient services into day surgeries, and explore other day surgeries. The notice encouraged qualified medical institutions to arrange some traditional inpatient diagnosis and treatment services during the day. Under the premise of ensuring medical quality and safety, they should set up day wards, and carry out daytime radiotherapy, chemotherapy, traditional Chinese medicine, neonatal daytime blue light treatment, and other medical services to improve the efficiency of medical services. In September 2021, the National Health Commission and the State Administration of Traditional Chinese Medicine jointly issued the “High-quality Development Promotion Action for Public Hospitals (2021–2025)“ [6], establishing and improving ten systems in the field of medical services such as appointment diagnosis and treatment, remote medical services, clinical pathway management, mutual recognition of inspection and testing results, medical social workers and volunteers, multi-disciplinary diagnosis and treatment, daytime medical services, rational drug management, high-quality nursing services, and satisfaction management. In November 2022, the National Health Commission issued the “Interim Provisions on Daytime Medical Quality Management in Medical Institutions”, which put forward basic requirements for the organization and operation management, quality control, and supervision and management of daytime medical services [7].

### Management model of day surgery

The management model of day surgery in China is a hospital-based platform management model under the unified management of the hospital and serving various clinical departments. The hospital formulates the management regulations and systems and supervises their operation. Currently, there are mainly three models: centralized treatment, decentralized treatment, and centralized decentralized treatment [8]. Centralized treatment refers to the medical institution having one or more independent day surgery wards or centers, with a dedicated medical team, serving patients in related clinical departments throughout the hospital to carry out day surgeries according to the unified hospital process. Representative hospitals include West China Hospital of Sichuan University [9] and Xiangya Hospital of Central South University. Decentralized treatment refers to the establishment of dedicated day surgery wards or beds in each clinical specialty ward, with a dedicated medical team to admit and manage day surgery patients following the unified hospital requirements for patients in their specialty. This model is more common in medical institutions that initially implement day surgery. Centralized decentralized treatment, also known as mixed treatment, refers to hospitals that have both centralized treatment and decentralized treatment models based on their conditions and characteristics of different specialties. This model diversifies the management of day surgery. Representative hospitals include Renji Hospital, affiliated with Shanghai Jiaotong University, and Beijing Tongren Hospital. These hospitals establish independent day surgery centers, with the head nurses responsible for daily activities. Each surgical department can also admit day surgery patients separately, admit patients according to the day surgery process, and have a dedicated person from the Medical Office to manage them. They also coordinate day surgeries in large operating rooms based on the overall resources of the hospital. From 2018 to 2019, the proportion of “decentralized treatment and unified management” decreased from 43.33 to 37.14%; the proportion of “mixed treatment and unified management” increased from 28.33 to 32.86%, and the proportion of “centralized treatment and unified management” increased from 25 to 25.7%. As the ambulatory surgery model has evolved, patient acceptance has also increased. In order to better cater to patients, hospitals are adjusting their patient management approach and are inclined towards establishing an ambulatory surgery center for integrated treatment and centralized management.

### Scope of day surgery

The scope of day surgery procedures is constantly expanding. In 2016, the National Health Commission recommended the first batch of day surgery procedures, with 43 items (statistically based on the Catalogue of National Clinical Version 2.0 of Operation Codes). In 2019, the second batch of day surgery procedures was recommended, with 77 items (statistically based on the Catalogue of National Clinical Version 2.0 of Operation Codes). In 2022, by integrating the first two batches of day surgery procedures, 708-day surgery procedures were recommended (statistically based on the Catalogue of National Clinical Version 3.0 of Operation Codes). The most frequently performed specialties are general surgery, ophthalmology, ENT, plastic surgery, and orthopedics. The coverage of day surgery procedures is gradually expanding, and the difficulty of surgery is gradually increasing. In 2012, the Notice of the General Office of the Ministry of Health on the issuance of graded management Measures for surgery in medical institutions (Trial) stipulated that surgical procedures are categorized into four levels based on their technical difficulty, complexity, and risk. Primary surgeries refer to common procedures with low risk, simple processes, and low technical difficulty. Secondary surgeries refer to procedures with moderate risk, general complexity, and technical difficulty. Tertiary surgeries refer to procedures with high risk, complex processes, and high technical difficulty. Grade 4 surgeries refer to major procedures with high risk, complexity, and technical difficulty. Some hospitals that started earlier have begun to explore day models for high-difficulty surgeries. For example, in 2018, the proportion of level III and IV surgeries in day surgeries at Renji Hospital in Shanghai reached 51.7%, and the proportion of level III and IV surgeries in day surgeries at Tongren Eye Hospital in Beijing reached 72.17%. According to the National Health Commission’s “2020 National Medical Service and Quality Safety Report”, the median proportion of level III and IV surgeries in day surgeries at tertiary medical institutions is 29.43% and 4.25% respectively [10].

### The payment method of medical insurance for day surgery

At present, 30 provinces have issued relevant documents on the medical insurance payment policy for day surgery. They are Beijing, Shanxi, Tianjin, Inner Mongolia, Hebei, Liaoning, Heilongjiang, Jilin, Zhejiang, Anhui, Jiangsu, Shandong, Fujian, Jiangxi, Guangdong, Guangxi, Hainan, Henan, Hubei, Hunan, Sichuan, Guizhou, Yunnan, Chongqing, Shaanxi, Qinghai, Xinjiang, Gansu and Ningxia. Among them, the provinces and cities with the most covered diseases are Changzhi City in Shanxi with 320 diseases, Shanxi with 200 diseases, Zhejiang with 158 diseases, and Chongqing with 120 diseases. The payment methods for day surgery are as follows: project payment in Shandong and Jilin; diagnosis-related group (DRG) payment in Zhejiang, Sanming City in Fujian Province, and Panzhihua City in Sichuan Province; payment by disease intensity points (DIP) in Zhongshan City in Guangdong and Zhengzhou City in Henan; and payment by disease type: more than 20 provinces and cities including Jiangsu, Beijing, Tianjin, and Shanxi. The medical insurance payment standard for day surgery is as follws: the cases included in the day surgery settlement management by Qingdao Municipal Health Security Bureau are regarded as ordinary inpatient cases and enjoy inpatient medical insurance treatment according to the regulations. The payment standard is the same as that of non-day inpatients; the Gansu Provincial Medical Security Service Center stipulates that when patients are discharged from the hospital for settlement, they shall be settled per the current medical insurance inpatient treatment policies. The starting standard shall be settled at 50% of the original standard, which shall not be accumulated or reduced. The medical insurance fund payment ratio of tertiary medical institutions is 82% of the total cost standard, and that of secondary medical institutions is 78% of the total cost standard; the payment standard of day surgery cases in Sanming City has been adjusted from 75 to 85% of the C-DRG group’s payment standard.

The scope of payment for daytime surgery packaging varies among medical insurance companies in different provinces. Beijing, Shandong, Liaoning, Jiangsu, Guangdong, Guangxi, etc. shall pay for pre-admission examination fees, in-hospital surgical fees, and post-discharge disposal fees; Hebei Province shall pay for the hospitalization expenses during daytime surgery and outpatient expenses for a total of no more than one week before and after hospitalization and discharge during daytime surgery; Guangdong Province shall pay for the medical expenses related to the 10 days before and 5 days after surgery for daytime surgical diseases; outpatient expenses during the hospitalization period of daytime surgery in Chongqing, as well as one week before hospitalization and one week after discharge for daytime surgery (in the same medical institution); related medical expenses incurred from the time of pre-hospitalization management to the end of clinical pathway in Mount Huangshan City, Anhui Province, are included in the medical insurance reimbursement settlement scope. Shanxi, Zhejiang, Fujian, Hunan, Qinghai, and other provinces pay for daytime surgical expenses, including pre-admission examination fees and in-hospital surgical expenses; Hainan, Shandong, and Hunan stipulate that the payment scope for daytime surgery is one week before surgery; Zhejiang and Fujian stipulate that the payment range for daytime surgery is two weeks before surgery; the payment scope for daytime surgery in Shanxi is relatively short, including the cost of preoperative examination for 3 days. The daytime medical insurance policies in various regions are still in the exploratory stage, with varying policies. In terms of the time limit for outpatient examination fees, there is a difference of 3 days to 2 weeks in the inclusion of hospitalization reimbursement in medical insurance in different regions [11].

The starting payment line standards for daytime surgery fees are the same as the starting payment standards for inpatient patients in different provinces and cities, with individual provinces and cities introducing incentive policies to promote the development of daytime surgery. Only expenses exceeding the starting payment line for medical insurance reimbursement will be covered by medical insurance, and expenses below the starting payment line must be paid by the insured individual themselves. In Chuxiong City, the starting payment line for daytime surgery medical insurance is 50% of that for non-daytime patients. In April 2018, the Human Resources and Social Security Bureau of Chuxiong Prefecture in Yunnan Province approved the request of the Chuxiong State Hospital to reduce the starting payment for daytime surgery diseases. The starting payment for urban workers’ medical insurance in Chuxiong was reduced from 1000 yuan to 500 yuan, and the starting payment for urban and rural residents’ medical insurance was reduced from 800 yuan to 400 yuan. The starting payment line for daytime surgery medical insurance in Hebei, Qinghai, Shanxi, Jilin, and other provinces and cities is not set, and in principle, it should be lower than the standard for inpatient treatment of the same disease type; Heilongjiang Province does not set a starting payment line, and daytime surgery includes the full cost of outpatient treatment. Other provinces and cities have the same starting payment line for medical insurance as inpatients.

## Day surgery conducted in a Grade III Class A ophthalmology hospital

According to the “Notice on the Trial Implementation of Basic Standards for Medical Institutions” issued by the Ministry of Health, hospitals are divided into general hospitals, traditional Chinese medicine hospitals, integrated traditional Chinese and Western medicine hospitals, specialized hospitals, etc. Specialized hospitals are divided into second-level and third-level specialized hospitals. Third-level specialized hospitals require a total of more than 150 hospital beds. They are also equipped with corresponding health technicians, clinical specialties, and medical technology departments. The requirements for annual discharge cases are more than 15,000, and the total number of operations is more than 10,000, of which the proportion of third and fourth-level operations is more than 40%. The requirements for second-level specialized hospitals will be correspondingly reduced.

Ophthalmic surgery is suitable for day surgery due to its “short, flat, and fast” characteristics. The hospital adopted the day surgery model in 2016, and from the initial decentralized treatment to the centralized treatment implemented in 2019, the management was centralized. The day surgery center is equipped with 60 beds, 8 operating rooms, and 20 nursing staff. In addition to corneal transplantation and patients with fundus diseases, various specialist diseases are treated in the day surgery area. The following is the operation volume of various surgical procedures for patients undergoing day surgery from 2019 to 2023 (Table 1).

**Table 1:** The number of eye surgeries (surgery eye times) for patients undergoing day surgery from 2019 to 2023

Disease name	Year 2019	Year 2020	Year 2021	Year 2022	Year 2023
Cataract surgery	2905	3765	4364	3844	5270
Pterygium excision	86	284	407	441	678
Strabismus surgery	110	222	469	343	649
Vitreous cavity injection	187	34	639	1018	1118
Eye, nose, and orbital surgery	4	0	76	91	164
Other items	20	113	67	85	201
Total	3312	4418	6022	5822	8080

Since May 2019, the daytime ward has been put into use, implementing centralized treatment and management. The Total in patient surgery volume refers to just patients who are admitted as in-patients and undergoes surgery. As such the total number of patients who underwent surgery for the period would be Day surgery volume + Total inpatient surgery volume. Day surgery accounts for an increasing proportion of elective hospitalizations year by year. According to statistics, in 2023, day surgery accounted for 70.38% of total hospitalizations (Table 2). According to statistics, the number of day surgery operations reached 8,080 in 2023, accounting for 70.38% of the total number of patients undergoing surgery. Table 2 shows the details. Day surgery plays a positive role in the rational use of medical resources, timely treatment of patients, doctor-patient relationship, and patient satisfaction rate, and has been widely recognized. This is confirmed by the data showing that the number of operations has increased year by year over the past five years.

**Table 2:** Percentage of day surgery and total patient (eye surgeries)

The specified year	Day surgery volume (eye surgeries)	Total patient operations (eye surgeries)	Proportion(100%)
Year 2019	3312	7471	44.33
Year 2020	4418	6816	64.81
Year 2021	6022	8990	66.98
Year 2022	5822	8448	68.91
Year 2023	8080	11,480	70.38

To make the day-surgery area of ophthalmology run efficiently, the hospital has developed the day-surgery patient inpatient process together with multi-departments and multi-disciplines, including the Medical Department, Nursing Department, Outpatient Department, clinical doctors, day-surgery area and so on. The whole process of the day-surgery information system covers the initial screening of patients in the outpatient department (this step is completed in the doctor’s outpatient clinic), pre-operative examination and testing, surgical evaluation and day-surgery inpatient application, anesthesia evaluation, day-surgery information verification, pre-admission education, surgical appointment, surgical confirmation, and other full process information management. The management system integrates HIS outpatient doctor stations, charging stations, nurse workstations, anesthesia systems, and inspection and testing systems through data interaction of the hospital information platform. Currently, it can dynamically realize patient information registration, medical history data, doctor’s orders inquiry, pre-operative evaluation, day-surgery bed reservation, pre-operative education, admission handling, cancellation handling, and other patient information extraction at each node to achieve various information interconnection and interworking of patients during day-surgery implementation. The outpatient information of patients is realized with the integration of the inpatient system. The examinations and medical history collected during the pre-hospitalization period can be associated with the inpatient information system after the patient is admitted to the hospital. After the patient is admitted to the hospital on the day of surgery, the clinical doctors and responsible nurses can import the information collected from the outpatient department into the inpatient medical records. In 10–15 min, they can complete the writing of inpatient medical records and nursing assessments, which greatly improves work efficiency. The first operation start rate in the operating room has increased from 78.9 to 90.6%. The medical team is highly integrated, efficient, and cooperative, with each performing their duties. This has greatly shortened the time patients stay in the hospital and preoperative waiting time, and improved work efficiency and patient satisfaction.

The implementation of day surgery effectively reduces medical costs and shortens the average length of hospital stay, achieving a win-win situation for medical insurance, hospitals, and patients.

**Table 3:** Inpatient expenses of a top-three hospital from 2019 to 2023

Project	Year 2019	Year 2020	Year 2021	Year 2022	Year 2023
Average hospitalization days (days)	2.04	1.4	1.44	1.41	1.41
Average hospitalization expense (yuan)	9147.18	8038.7	8161.42	7465.26	7380.98
Turnover times of beds	86.03	80.78	104.85	95.75	131.13

After the hospital’s day surgery area was put into use, the average hospitalization cost of the two wards showed a significant downward trend. As shown in Table 3, the average hospitalization cost decreased from 9147.18 yuan per person in 2019 to 7380.98 yuan per person in 2023, offering patients genuine cost savings on medical expenses. The table also shows that the average length of hospital stays decreased from 2.04 days to 1.41 days, reducing the time patients spent in the hospital and potentially reducing direct non-medical costs.

Due to the large proportion of cataract patients in the annual number of day surgery procedures, exceeding 65% (Table 1), this study takes cataract surgery as an example to collect various data for analysis. Using SPSS 26.0 software to establish a database and process data, a chi-square test and a non-parametric rank sum test were conducted to collect information on discharged patients with cataract from day and non-day wards in 2021–2022, including regional distribution, length of stay, hospitalization costs, etc. The differences in direct medical costs, direct non-medical costs, indirect costs, and total costs of cataract surgery in day and non-day wards were compared.

2.4.1 Table 4 shows that cataract patients are generally older, and surgical patients are mainly concentrated in those over 60 years old. The average hospitalization days for day surgery are significantly lower than those for non-day cataract surgery, and the main payment method is mainly medical insurance surgery.

**Table 4:** Patient data between 2021and 2022

Item	Day-ward group(n = 6645)	Inpatient group(n = 268)	p-value
Gender (cases (%))			
Male	2542(38.25)	116(43.28)	0.097
Female	4103(61.75)	152(56.72)	
Age (years, M (P25, P75))	70 (64,77)	68 (60,77)	< 0.001
Mean length of hospitalization (days, (P25, P75))	0.2(0.18,0.23)	1.2(0.9,1.93)	< 0.001
Medical insurance payment type(cases (%) )			
Provincial-level and Hangzhou city medical insurance	4785(72.01)	102(38.05)	< 0.001
Medical insurance in different provinces	1572(23.66)	100(37.31)	
Self-paying	288(4.33)	66(24.63)	
Patient regional distribution (cases (%))			
Patients from Hangzhou	5410(81.41)	167(62.31)	< 0.001
Patients from Zhejiang province excluding Hangzhou (including patients from outside Zhejiang province)	1235(18.59)	101(37.69)	

The direct non-medical costs are calculated based on the meal and transportation expenses paid by the patient and their accompanying family members during the hospitalization period. Assuming one family member accompanies, according to the statistics of the Hangzhou Municipal Bureau of Statistics, the per capita consumption expenditure of urban residents in 2021 was 48,629 yuan [12], with an average daily expenditure of 133 yuan. Therefore, the total daily direct non-medical cost for the patient and family members was calculated as 266 yuan. Indirect costs referred to the loss of work due to the absence of family members accompanying the patient during hospitalization (since the patients were generally over 60 years old, the indirect costs for the patients themselves were not calculated). The indirect cost was calculated using the formula: Wage loss Hospitalization days of the patient’s Daily monetary salary. According to the data from the Hangzhou Municipal Bureau of Statistics, the average annual salary of employees in private units in Hangzhou in 2021 was 84,906 yuan [13], with an average daily salary of 340 yuan. In addition, other intangible costs were difficult to quantify and were not considered for the time being. As shown in Table 5, the direct medical cost, direct non-medical cost, indirect cost, and total cost of daytime surgery after lens removal were lower than those of hospitalized patients, and this difference was statistically significant (*P* < 0.05). This conclusion is consistent with that of Zhang Yidan et al., who found that daytime surgery can reduce patients’ invisible social cost expenditure [14].

**Table 5:** Cost comparison of day-ward group and inpatient group Yuan, M (P25, P75)

Group	Direct medical cost after removing the intraocular lens	Indirect costs	Direct non-medical costs	Total cost
Day-ward group	4503.67(4291.15,4830.8)	69.49(61.66,79.83)	54.36(48.24,62.46)	6812.84(6067.3,8528.86)
Inpatient group	4747.30(4389.36,5250.32)	414.09(317.46,654.77)	323.97(248.37,512.26)	7531.25(6696.26,8704.25)
z-value	-8.151	-23.911	-23.911	-7.102
p-value	< 0.001	< 0.001	< 0.001	< 0.001

### Analysis of patients’ self-paid medical burden under day surgery operation

From the perspective of patients’ out-of-pocket payments and medical insurance fund bearing medical expenses during the day ( Table 6), the proportion of out-of-pocket payments for provincial and Hangzhou medical insurance patients was 21.3%, far lower than the 43.77% for provincial non-local medical insurance patients. This indicates that due to policy advantages and the absence of cross-regional medical treatment issues, the reimbursement proportion for provincial and Hangzhou medical insurance patients was high, and their economic burden was light.

**Table 6:** Day ward medical insurance patient self-payment ratio (2021 to 2022)

Type of medical insurance	Out-of-pocket ratio
Zhejiang Municipal Medical insurance	21.3% (0.49%,30.32%)
Zhejiang out-of-province medical insurance	43.77% (18.88%,59.2%)
z-value	-29.415
p-value	< 0.001

### Evaluation of therapeutic effect in daytime wards

The rate of an unplanned second surgery and the satisfaction rate of the 6,913 patients undergoing day surgery between 2021 and 2022 were collected using telephone and outpatient follow-up, review of medical records, data extracted from the HIS system, and the distribution of questionnaires. The results of this analysis are shown in Table 7.

Unplanned second surgery refers to further unscheduled surgeries due to complications caused by the surgery or insufficient medical care. In this study, the unplanned second surgery rate = the number of unplanned second surgeries/the number of surgeries × 100%.

Patient satisfaction was evaluated by a third-party questionnaire that included 20 items from 5 perspectives, namely, day-ward medical procedures, day surgery treatment effect, attitudes of medical staff, doctor-patient communication, and health education. The items were graded with “very satisfied (score 5)”, “satisfied (score 4)”, “OK (score 3)”, “dissatisfied (score 2)”, and “very dissatisfied (score 1)”. The total questionnaire score could vary between 20 and 100, with a total score ≥ 90, meaning that the patient felt satisfied. In this study, the satisfactory rate = the number of cases of total score ≥ 90/total number of cases × 100%.

**Table 7:** Unscheduled second surgery rate and patient satisfaction rate in the day ward (2021 to 2022)

Year	Cases	Unscheduled second surgery rate /%	Patient satisfaction rate /%
2021	3668	0.05	99.1
2022	2977	0.1	99.5

## Discussions and recommendations

### The promotion of the day surgery service model is an inevitable trend for the efficient utilization of medical resources

China’s day surgery is developing safely and steadily with the pace of exploration, pilot implementation, and gradual expansion. With the continuous improvement of medical technology, the medical service model is gradually changing, and day surgery is an outstanding performance of this transformation. Day surgery helps to improve the efficiency of hospital operations, reduce patient medical expenses, shorten visit time, and improve patient satisfaction [15–17]. The practice and development status of day surgery in China in the past 20 years show that China needs to increase the proportion of day surgery. According to the 2020 National Health Commission statistics, the number of hospitals that have carried out day surgery has reached 2409, and the total number of cases of day surgery carried out each year has reached 1172.82 million cases. Day surgery accounts for 17.6% of total surgical cases, and 70% of tertiary hospitals nationwide have carried out day surgery. Currently, the top hospitals in China that carry out day surgery include Renji Hospital Affiliated with Shanghai Jiao Tong University School of Medicine, which accounts for more than 30% of total surgical cases. However, there is still a large distance compared with developed countries in Europe and America, where day surgery accounts for more than 70%. The ophthalmology specialty hospital Zhongshan Ophthalmic Center, Sun Yat-sen University, has achieved a 93% proportion of day surgery cases in its total surgical cases, and Beijing Tongren Hospital affiliated to Capital Medical University has reached a 90% proportion of day surgery cases in its total surgical cases. Day surgery at the author’s hospital accounts for about 70% of total surgical cases. Compared with benchmark hospitals, this hospital still has considerable room for development. Because ophthalmic surgery has the characteristics of being “short, flat, and fast” suitable for day surgery, it has been recognized by everyone.

The results of the seventh national census in 2021 show that the proportion of people aged 60 and over in China has reached 18%. The aging population trend has exacerbated the shortage of high-quality medical resources. How can the utilization efficiency of high-quality medical resources be improved and the capacity of medical services be expanded? Based on the improvement of minimally invasive surgical technology service capabilities, the development of anesthesia and postoperative pain management, and the increased acceptance of day surgery service models by patient populations, they should accelerate the promotion of public hospital day surgery service models, gradually narrowing the gap with developed countries in Europe and America. At the same time, they should sink technology, improve medical standards, and expand the authority of non-tertiary hospitals for day surgery, so that more patients can benefit.

### Perfecting medical insurance policy to promote the smooth development of day surgery

China’s medical insurance payment system is divided into outpatient and inpatient, with a focus on supporting inpatient costs. Some forms of medical insurance, such as the new type of urban-rural cooperative medical care, do not support reimbursement for outpatient services in tertiary hospitals, and the promotion of commercial medical insurance in China is not high, with a Low proportion of insurance coverage. It affects the willingness of some patients to implement day surgery [11]. Currently, the medical insurance management policies for day surgery in various regions of China have not been unified, and the total reimbursement ratio for day surgery in many regions is low. Many diseases that could be included in day surgery are still managed as elective surgeries. Some medical insurance departments believe that day surgery cannot be “opened” at will, and needs to be strictly evaluated and calculated. If simple outpatient surgeries are mixed into day surgery, it will not save costs, but rather waste medical insurance funds. The National Health Department has updated the recommended day-case disease catalog multiple times, but the medical insurance department still does not support expanding the scope of medical insurance day-case diseases. Day surgery service processes include pre-hospital appointment and evaluation, in-hospital treatment, and post-hospital follow-up and rehabilitation stages. Although some regions have implemented bundled payments for day surgery, the current medical insurance policies for day surgery across the country are still in the exploratory stage, with different policies and an urgent need for further experience summarization and unified standards at the national level.

### Establishing day surgery quality management standards to ensure medical quality and safety

In November 2022, the National Health Commission issued the Interim Provisions on the Management of Daytime Medical Services in Medical Institutions [8], which clearly states that medical institutions should strengthen the management of daytime medical diseases and technologies. Following the principles of science, safety, and standardization, medical institutions should formulate their own daytime disease and technical catalogues and implement dynamic management. To improve the level of medical quality and safety, medical quality and safety management should be integrated into the construction of specialized capacity, and scientific management should be carried out using medical quality management tools to strengthen the application of quality control indicators and the collection, analysis, and feedback of medical quality and safety data, and carry out targeted improvement based on the evidence of medical quality and safety. To ensure the quality and safety of day surgery patient services, further research is needed on a series of standardized norms and systems, including the admission standards for doctors, patients, diseases, and surgical procedures, the standardization of medical record writing, anesthesia specifications, quality monitoring indicators, emergency plans, follow-up norms, community referral models for postoperative rehabilitation, and the establishment of a homogenized day surgery quality and safety evaluation system. Establishing day surgery quality management norms from system construction, management mechanisms, operation processes, and quality monitoring will comprehensively promote the healthy, orderly, and standardized development of day surgery.

### Improve the whole process management of day surgery based on information support

Medical institutions that carry out day surgery are constantly improving their day surgery management information systems, allowing patients to travel less and information to travel more. Through the improvement of the information management system, the service process of day surgery is optimized, service efficiency is improved, and modern hospital management is realized through information technology. The hospital uses cloud computing, artificial intelligence, and face recognition information technology to build a day surgery pre-hospitalization management system, an Internet ward platform, a follow-up management platform, a day surgery electronic medical record, and a day surgery process-oriented specialist nursing robot to reduce the rate of missed appointments for day surgery, unplanned secondary surgery, and unplanned readmission, and improve patient satisfaction and sense of gain [18, 19]. The day ward bed automatic allocation system, surgery scheduling system, day medical quality monitoring system, and establishment of hospital-community tracking follow-up management database of Renji Hospital affiliated with Shanghai Jiaotong University School of Medicine have achieved good social benefits.The Day Surgery Center at West China Hospital, Sichuan University has improved the utilization of operating rooms by further refining the day surgery process and using the JSSP model to allocate resources involved in various processes of surgery. It also manages anesthesiologists, surgeons, anesthetic resuscitation doctors, and other hospital resources [20]. Job Shop scheduling problem (JSSP) is a class of combinatorial optimum problems with time, sequence and resource constraints.

## Conclusions

The implementation of the day surgery model has achieved certain results. It has optimized the surgical process, enabling more patients to receive surgical treatment in a timely manner and alleviating the tension of medical resources. It reduces the contact time between patients and the hospital, lowers the risk of nosocomial infection, and alleviates the psychological and financial burden of patients. Research has shown that under the premise of strictly controlling surgical indications and patient selection, the quality and safety of day surgery are comparable to traditional inpatient surgery. This further demonstrates the effectiveness of day surgery in ensuring medical quality and safety.

Despite the numerous advantages of day surgery, it does not mean that all surgeries are suitable for this model. Specific surgeries still need to be comprehensively evaluated based on the patient’s condition, the type of surgery, and the actual situation of the hospital. Due to differences in medical standards, patients prefer to travel to hospitals with higher levels of technology for surgery, resulting in inconvenience for patients and their families. Therefore, it is necessary to actively explore the mode of rehabilitation in the community after day surgery. In addition, for patients who travel for medical treatment, most regions require a certain percentage of self-payment before reimbursement, which is lower than the reimbursement rate for local medical treatment. This part of patients will bear higher costs on their own. With the promotion of disease-related group payment and the encouragement of an outpatient coordination system in China, it is important to explore a medical insurance payment model that adapts to the implementation of day surgery, breaks down barriers between provinces and cities, plays its economic leverage role, and guides patients to accept the day surgery model.

## Data Availability

The datasets used and/or analyzed during the current study are available from the corresponding author upon reasonable request.
